# Internet of things in healthcare for patient safety: an empirical study

**DOI:** 10.1186/s12913-022-07620-3

**Published:** 2022-03-01

**Authors:** Tahera Yesmin, Michael W. Carter, Aviv S. Gladman

**Affiliations:** 1 Center for Healthcare Engineering, Department of Mechanical and Industrial Engineering, University of Toronto, Toronto, Canada; 2 Chief Information Officer and Chief Medical Information Officer, Mackenzie Health, Toronto, Ontario Canada

**Keywords:** Internet of things, Quality of care, Patient safety, Patient falls, Hand hygiene, Staff experiences

## Abstract

**Introduction:**

This study evaluates the impact of an Internet of Things (IoT) intervention in a hospital unit and provides empirical evidence on the effects of smart technologies on patient safety (patient falls and hand hygiene compliance rate) and staff experiences.

**Method:**

We have conducted a post-intervention analysis of hand hygiene (HH) compliance rate, and a pre-and post-intervention interrupted time-series (ITS) analysis of the patient falls rates. Lastly, we investigated staff experiences by conducting semi-structured open-ended interviews based on Roger’s Diffusion of Innovation Theory.

**Results:**

The results showed that (i) there was no statistically significant change in the mean patient fall rates. ITS analysis revealed non-significant incremental changes in mean patient falls (− 0.14 falls/quarter/1000 patient-days). (ii) HH compliance rates were observed to increase in the first year then decrease in the second year for all staff types and room types. (iii) qualitative interviews with the nurses reported improvement in direct patient care time, and a reduced number of patient falls.

**Conclusion:**

This study provides empirical evidence of some positive changes in the outcome variables of interest and the interviews with the staff of that unit reported similar results as well. Notably, our observations identified behavioral and environmental issues as being particularly important for ensuring success during an IoT innovation implementation within a hospital setting.

## Background

Technology has come to be employed throughout the healthcare sector to improve patient outcomes and safety while reducing costs and optimizing resource utilization [1]. The International Telecommunication Union (ITU) projects that the Internet of Things (IoT)—alongside developments in item identification, wireless sensor networks, and embedded systems—will soon connect the world’s many devices in a sensory, intelligent manner [2, 3]. The Federal Trade Commission (FTC) states IoT as “an interconnected environment where all manner of objects have a digital presence and the ability to communicate with other objects and people” [4]. Though the term IoT was first introduced and defined by Kevin Ashton in 1999 as a network of uniquely addressable and interoperable objects with radio-frequency identification (RFID) technology [1], gradually the modern IoT platform has empowered a steadily increasing number of connected devices, including RFID tags, mobile phones, and actuators to communicate through embedded sensors and relay enormous amounts of data with little to no human interaction [5, 6]. These data can then be collected, recorded, and analyzed to improve the care-delivery process. While IoT is a relatively new concept in healthcare, it has long been employed in agriculture, environmental monitoring, food processing, smart grids, traffic management, home automation, firefighting, and mining [7–10]. While there are certainly technological challenges regarding privacy, trust, and security in the application of IoT in healthcare [11, 12], many studies have explained the working principles of IoT and emphasized that IoT could transform healthcare [4, 13, 14].

Patient safety, one of the six elements of quality of care, is defined as avoiding injuries to patients while implementing care aimed at helping them [15]. The World Health Organization defines patient safety as the prevention of errors and adverse effects on patients associated with health care [16]. Globally, adverse events stemming from a lack of patient safety constitute one of the ten leading causes of death and disability; in high-income countries, 50% of these events can be prevented [16]. According to Morris and O’Riordan (2017), among these adverse events, inpatient falls constitute the most frequent reported safety incident in National Health Service (NHS) hospitals; Agency for Healthcare Research and Quality (AHRQ) reports that 700,000 to one million hospitalized patients experience falls each year [17]. Another report details the fall rate as 3–5 per 1000 bed-days; about one-third of these falls result in injuries, such as head trauma and fractures [18]. Healthcare-associated infections (HCAIs) constitute another major adverse event that stems from unsafe care. Nearly 1.7 million hospitalized patients acquire HCAIs while receiving treatments [19], and several studies have shown that improved hand hygiene can significantly mitigate these infections (by around 50%) [20–22].

Numerous interventions have been widely applied in hospital settings to improve patient safety. One such method entails the promotion of a culture of safety; another involves improvements to specific aspects of care delivery that staff identify as harmful to patients [23]. Several studies have assessed the recent trend of utilizing IoT in healthcare to improve patient safety [24, 25]. Ahmadi et al. (2019) assert that IoT can be used to achieve several goals in hospital management, including preventing infections [1]. However, despite its various applications in patient safety, IoT still requires more research and experimentation, as most existing research is in the early stage of testing new methodologies [25]. Studies on the practical impacts of IoT on patient safety measures in a hospital setting are sparse. Thus, this study aims to explore an advanced IoT-based intervention in a hospital setting and empirically demonstrate its impact on patient safety. This research contributes real-life, application-based evidence to validate the claims in the literature that IoT improves patient safety by showing its impact on patient-fall and hygiene-compliance rates [24, 25]. We use both pre-post and time-series analyses to demonstrate IoT’s impact on patient safety. Section 2 provides a comprehensive literature review on the application of IoT to prevent patient falls and improve hand hygiene. Section 3 details the methods used in this study. Section 4 reports our results, and Section 5 offers a discussion and some conclusions.

## Related works

### Internet of things in healthcare

The application of IoT in medical fields is consistently expanding. In 2014, Xu et al. conducted a survey to provide a detailed review of the IoT architecture (alongside other key technologies) that is revolutionizing healthcare [8]. In 2015, Islam et al. surveyed the working methodologies and use of IoT in healthcare, considering various IoT services and applications for both single and clustered medical conditions [26]. Another review noted that IoT can empower individuals by providing cost-effective and personalized care in both clinical care (for in-patients) and remote monitoring [27]. One study assessed the underlying architecture of recent IoT applications in healthcare, such as a smart pill that measures medical adherence, ambient assisted living for elderly patients, and interactive m-health for diabetic patients [28]. Dimitrov detailed how the pharma industry partners with the tech industry to develop IoT-based healthcare systems that improve patient care [29].

While many researchers focus on the architecture and development of various IoT-based healthcare applications, some studies focus superficially on how IoT impacts certain aspects of hospital environments, such as patient safety and work efficiency. Many studies assert that IoT can significantly improve patient safety, as various alarm systems can alert care providers to evolving patient conditions, enabling them to act accordingly [30–32].

### Internet of things and patient falls

IoT can be applied to reduce patient falls in both hospital and home settings. Kang et al. mentioned that achieving patient safety by reducing patient falls is a very significant application of IoT in hospitals [32]. Several studies have proposed IoT-based fall-reduction systems, most of which involve two device types: wearable devices and external systems [33]. In 2016, Vaziri et al. proposed a system to assess a person’s at-home falling risk and deliver a tailored exercise and fall-prevention program [34]. In 2017, Joshi and Nalbalwar proposed a vision-based fall detection and alert system that uses a single camera to detect features like orientation angle, aspect ratio, center of mass, and Hu moment invariants (calculated from the white pixels extracted from the silhouette of the foreground objects) to detect, document, and alert people to falls [35]. One study detailed a fall-detection system that provides a centralized system through a mobile application based on the cloud to gather data on all monitored persons [36]. In 2019, Yee et al. developed a wearable, sensor-based fall-prevention device that can differentiate between falling and non-falling cases with the help of a k-NN classifier [37]. In another 2019 study, Khan et al. proposed a wearable device consisting of a camera, gyroscope, and accelerometer that remotely detects patient falls [38]. Similarly, textile-based systems have also been proposed by other researchers [39, 40].

Though many studies have proposed new fall-detection approaches, few have included empirical or intervention-based studies in a hospital setting to assess their performance. Vaziri et al. (2016) reported on the user-experience and user-acceptance aspects of their iStoppFalls system among older adults [34]. Another evaluation was conducted by Balaguera et al. (2017) in a medical-surgical unit of a teaching hospital, where a sensor pad was placed between the mattress and bedsheet of the recruited patients. Their study reported no bed falls over 234 patient days following this system’s implementation among 91 patients. Nursing staff responded to alerts from the fall-prevention system on their mobile phones in an average of 45.9 s. Though this study compared the post-intervention fall rate with the pre-intervention fall rate, it did not consider all of the unit’s patient types [41]. Table 1 presents a list of articles that applied IoT to detect or prevent patient falls, where ‘Yes’ indicates the presence and ‘No’ indicates the absence of the mentioned attributes in that tables from that study.

**Table 1:** Application of IoT to detect or prevent patient falls

Authors	Methodology-based		Intervention-based/Trial-based	
	Wearable	External	Hospital	Home
Varizi et al., 2016 [34]	No	Yes	No	Yes
Joshi & Nalbalwar, 2017 [35]	No	Yes	No	No
Mrozek, Koczur, & Małysiak-Mrozek, 2020 [36]	No	Yes	No	No
Yee et al., 2019 [37]	Yes	No	No	No
Khan et al., [38]	Yes	No	No	No
Niazmand, Jehle, D’Angelo, & Lueth, 2010 [39]	Yes	No	No	No
Mezghani, Ouakrim, Islam, Yared, & Abdulrazak, 2017 [40]	Yes	No	No	No
Balaguera et al., 2017 [41]	No	No	Yes	No

### Internet of things and hand-hygiene compliance

As hand-hygiene (HH) compliance is one of the most important factors to reduce hospital-acquired infections, accurate measurement of HH compliance among healthcare providers is a vital element of high-quality care delivery. HH compliance is mainly measured manually by hospital auditors. However, studies suggest that the Hawthorne effect—the change in providers’ behavior when they are aware of being monitored—challenges the value of human auditing and encourages the use of automatic HH-measuring devices. Therefore, IoT has great potential to improve HH compliance. Research has shown that one automatic HH-monitoring system can precisely identify the times and locations of hand washing. However, it could not aid in a badge-based system, as it failed to recognize which individuals were cleaning their hands [42]. In another retrospective study, Xu et al. (2021) measured the effectiveness of an IoT-based management system on HH compliance in an intensive care unit. They found that the new system increased the HH compliance rate among all staff aside from the cleaners [43]. Similarly, in another study, an IoT-based automatic monitoring system was implemented to collect real-time data and employ gamification to improve HH compliance among nurses in both simulation and clinical environments. While the simulation setting revealed a 100% HH-compliance rate, the nurses showed little interest in considering badges for future improvement [44].

### Interrupted time series analysis (ITS) with segmented regression

Segmented regression is a statistical method that is widely used to estimate intervention effects in ITS studies. Many researchers have used this quasi-experimental method to evaluate intervention impacts [45]. Wagner, Soumerai, Zhang, and Ross-Degnan (2002) used this method to evaluate an intervention aimed at improving the quality of medication consumption [46]. In another study, researchers conducted a segmented regression analysis of a four-year interrupted time series to identify the impact of a policy intervention aimed at reducing the inappropriate use of key antibiotics. Their analysis revealed a significant decrease in total use and cost in the two years following the intervention [47]. Similarly, segmented regression for ITS studies was used in many other healthcare interventions, including to assess incremental costs [48], evaluate screening effectiveness [49], and evaluate new strategic interventions [50].

### Diffusion of innovation theory

We have designed our interview questionnaire based on Roger’s Diffusion of Innovation Theory, which is extensively used by the researchers to design studies that report user experiences. Based on this theory, there are four main determinants of the success of an innovation: communication channels, the attributes of the innovation, the characteristics of the adopters, and the social system. The attributes of innovation include five user-perceived qualities, which are relative advantage, complexity, compatibility, observability, and trialability [51]. Researchers used these five elements in their study to identify the justification of some of the clinical behaviors [52]e, to analyze nurses’ perceptions toward using a computerized care plan system [53], and to understand the factors impacting the use and patient acceptance of consumer e-health innovation [54].

## Methodology

### Study setting

This study was conducted at Mackenzie Health (MH) in Ontario, Canada, which has been identified as a leader of smart hospitals [55]. A pioneer in change, MH has implemented the data-driven concept of IoT in one of their strongest care units: the Mackenzie Health Innovation Unit (MHIU) at their Mackenzie Richmond Hill Hospital (MRHH) site. Established in June 2014, the MHIU has integrated modern technologies to improve the quality and efficiency of care while limiting costs.

#### Mackenzie health innovation unit: internet of things in healthcare intervention

The MHIU is a first-in-Canada hospital ward with 17 rooms (34 beds) that has embraced IoT to develop safe and efficient care delivery while continually producing real data [56]. These real data enable the intelligent evolution of care delivery over time. The applied technologies are as follows:

*Smart patient beds:* Smart beds are implemented to support safety protocols for at-risk patients by reducing harmful events, such as patient falls. Caregivers set the side rails, brakes, and safety alarms before leaving a patient’s room, and the bed (i) notifies caregivers if the patients leave their bed through the “bed exit alarm,” (ii) reminds caregivers to shift the patients’ positions to avoid bed sores through a “patient turn frequency reminder,” (iii) prevents a false bed exit alarm through awareness of when a nurse is in close proximity, and (iv) facilitates patient requests through an integrated call-bell system. This call bell consists of three call-type buttons—normal, pain, and bathroom and has a speaker that allows nurses to remotely communicate with their patients.

Smart beds, though similar in appearance to normal beds, have several sensors to collect and transmit data to a centralized server. They usually collect the following information:

- Guardrail status: It is important to know whether the guardrails are lowered or raised to prevent falls.
- Patient weight: The bed has pressure sensors to ensure accurate and timely weight measurements, avoiding lifting-related injuries among caregivers.
- Bed angle: The elevation of the head of the bed is important for patients with respiratory difficulties.

*Smart Hand Hygiene support solutions:* Through proximity RFID sensors, staff are monitored for HH practices and are alerted if they forget to wash their hands. HH stations are situated at the entrance and inside of each patient’s room. The HH system records whenever a caregiver uses it or misses an opportunity to clean (e.g., if a staff member enters the patient room without using the HH system). The sensors collect data on two HH moments (before entering and after exiting a room), handwashing locations, and room numbers.

*Smart badges:* Mackenzie Health assigns RFID badges to caregivers that identify their location. This enables the system to transfer patient calls to the nearest caregiver. Sensors are placed throughout the unit to quickly capture accurate caregiver locations. These badges also enable communication between staff and allow for rapid caregiver response times.

*Dome light indicators:* Installed outside (above the door) of each patient’s room and synced with the call-bell system, dome light indicators clearly show when a patient is at high fall risk. The status board lights up when a patient has called for assistance and brightly displays an “N” symbol when a caregiver is present in the room.

*Wall-mounted call stations and mobile phones:* Mackenzie Health has wall-mounted call stations and mobile phones to enable nurses to easily receive calls from patients. Each wall-mounted call station is a static touch-screen device that enables caregivers to answer, hold, or dismiss patient calls, displaying patients’ names, call type, patient room, and bed number. The stations are associated with the unit’s RFID location system. This advanced feature transmits patients’ calls directly to the call station closest to the assigned nurse of the calling patient. If a nurse moves before answering the call, the call is routed to the new nearest station. This system also enables nurses to call one another by viewing their location and making direct calls. The MHIU also provides nurses with mobile phones to accelerate processes pertaining to patient scans and diagnostic tests.

*Smart stations:* Smart stations are mounted in every patient room and provide specific patient information to their caregiver whenever necessary. This enables caregivers to respond quickly to the needs and requirements of the patients.

MHIU was intended to tests the benefits of IoT before applying these new technologies across their MRHH site and to their newly built hospital named Cortellucci Vaughan Hospital.

### Quality-of-care dimensions: patient safety

According to the existing literature, patient safety is fairly sensitive to IoT technologies [57]. As noted in the literature review, indicators of patient safety such as patient falls and HH compliance have been thoroughly investigated. In line with existing research, this study examines the following outcome indicators [58]:

(i) Patient-fall rate: A patient fall is defined as an event that results in a person inadvertently coming to rest on the ground or floor [18]. The patient-fall rate is measured as [58]:

$$\left(\frac{\mathrm{number}\ \mathrm{of}\ \mathrm{patient}\ \mathrm{falls}\ }{\mathrm{number}\ \mathrm{of}\ \mathrm{patient}\ \mathrm{days}\ }x\ 1000\right)$$

(ii) The HH-compliance rate is measured as [59]:

$$\frac{\begin{array}{c}\mathrm{Sum}\ \mathrm{of}\ \mathrm{the}\ \mathrm{number}\ \mathrm{of}\ \mathrm{times}\ \mathrm{HH}\ \mathrm{was}\ \mathrm{performed}\ \mathrm{for}\ \mathrm{all}\ \mathrm{HCPs}\ \\ {}\ \mathrm{before}/\mathrm{after}\ \mathrm{patient}'\mathrm{s}\ \mathrm{environment}\ \mathrm{contact}\end{array}}{\begin{array}{c}\mathrm{Sum}\ \mathrm{of}\ \mathrm{the}\ \mathrm{number}\ \mathrm{of}\ \mathrm{observed}\ \mathrm{HH}\ \mathrm{indications}\ \mathrm{for}\ \mathrm{all}\ \mathrm{HCPs}\ \\ {}\mathrm{before}/\mathrm{after}\ \mathrm{patient}'\mathrm{s}\ \mathrm{environment}\ \mathrm{contact}\end{array}}\ x\ 100$$

(iii) Staff experience: In addition to these outcome variables, existing research has indicated that staff experience during similar interventions has been important for perceived success among the users [22]. Therefore, we investigate staff experiences through qualitative semi-structured interviews. We received research ethics board’s approval to perform the interviews and all the interview methods were conducted in accordance with the relevant guidelines and regulations.

### Pre-post intervention study

We conducted a pre-post intervention study to evaluate the outcome of IoT implementation in the MHIU. We analyzed two indicators of patient safety, patient falls and HH compliance, using various statistical measures, including t-tests, chi-square tests [60], Mann-Whitney tests, and interrupted time series (ITS) analysis with segmented regression [45, 46]. Data collected between 2012 and 2016 were used to conduct a pre-post time series analysis of patient-focused indicators (patient falls) and a post-intervention analysis of staff-focused indicators (HH compliance). Since the MHIU is the only unit to have undergone this intervention, we could not find another unit or hospital implementing these technologies to use as our control group. However, there is a distinct date on which the MHIU implemented IoT, enabling sound pre-intervention and post-intervention interrupted time series analysis [61]. Additionally, we analyzed staff experiences by conducting semi-structured qualitative interviews using Roger’s Diffusion of Innovation Theory [51].

### Interrupted time series analysis with segmented regression

We analyzed the relationship between intervention and outcome using segmented regression, which is widely used when different timeframes are used as segments. We used the following formula to identify the impact of the intervention on patient falls [46]:

$${Y}_t={\beta}_o+{\beta}_1\ast {time}_t+{\beta}_2\ast {intervention}_t+{\beta}_3\ast post- slope/ time\ after\ the\ {intervention}_t+{\varepsilon}_t$$

**Where i.** Y_t_ is the mean outcome at time *t*, **ii.** *β*_o_ is the baseline level of the outcome at time zero, **iii.** *β*_1_ estimates the change in the mean outcome in unit time increase before the intervention (i.e., the baseline trend), **iv.** *β*_2_ estimates the level change in the mean outcome immediately after the intervention, and **v.** *β*_3_ shows the slope change following the intervention (post-intervention slope/time is a scaled interaction term to identify the effect of the time after the intervention). The sum of *β*_3_ and *β*_1_ is the post-intervention slope [61].

### Staff experience based on diffusion of innovation theory

We have conducted semi-structured, one-on-one, in-depth [62] interviews with the staff of MHIU to document their perspectives regarding the intervention’s effectiveness. The research objective of the interviews was to evaluate the MHIU before and after the intervention and identify the recommendations for the future. We created our questionnaire from Roger’s Diffusion of Innovation Theory, including five questions based mainly on (i) relative advantage (what the nurses experienced following implementation), (ii) compatibility of new technologies in their daily work, (iii) complexity, if any, (iv) trialability (problems faced following the implementation), and (v) observable results (changes observed as a result of the implementation). We also had a separate section to ask some demographic questions on gender and age.

Participant inclusion criteria of this interview were: (i) at least one year of full-time work experience at MH-so that they are well aware of the changes of the innovation unit, (ii) experience working for M and one other unit in the hospital- thus they can compare and report the changes of this unit, and (iii) expertise in using all technologies implemented in this unit- this will enable them to demonstrate all the aspects of the requested queries during the interview.

As this was only one unit of the hospital, considering that the total number of nurses work in this unit who meet the inclusion criteria we have interviewed 5 nurses- 3 Registered Practical Nurses (RPN) and 2 Registered Nurses (RN) for 30 min. An information document on the intervention and purpose of the interview and consent form for signature was provided to the nurses beforehand of the interview.

### Data collection and preparation

This study also used data on the patients’ age, gender, falls, and staff HH compliance. Though the MHIU was established in June 2014, the IoT technology was not fully functional until August 2014; therefore, data collected prior to August 2014 were analyzed as pre-intervention data, and data collected after August 2014 were analyzed as post-intervention data.

#### Patient falls

Data on patient falls from January 2012 to October 2016 were collected to conduct this study. Incident reports contained information on age, gender, time of fall, and type of fall.

#### Hand-hygiene compliance

Data from September 2014 to October 2016 on two moments of HH (before entering and after exiting a room), station type, and staff type were collected by sensors situated on each room’s HH system. These data contained a serial number, staff type, room number, and timestamp for each instance in which someone cleaned their hands. While analyzing more than one million rows of information on HH, we performed rigorous data cleaning. Data with any of the following three irregularities were deleted from the dataset:

(i) There should always be paired information on HH upon a caregiver’s entrance into and exit from a room. However, we noticed that, sometimes, there was multiple (usually 2) entrance information at the same time for a single room for the same person. In such cases, we deleted all but one entry.

(ii) Sometimes, HH information on the time of exit has been reported as an entry. As we cannot confirm the actual reason for such entry reports, we deleted these types of data.

(iii) When collecting HH information from sensors in a particular room, we found inputs from different rooms with missing information on the entrance or exit moments. For example, Room 0011 was present in the set of data for room 1133, and we could not find any explanation for this type of behavior. Such information was deleted from the dataset.

#### Staff experience

Nurses’ responses during these 30-min interviews were recorded on a laptop. Later, each interview was transcribed manually to a word processing document. The transcribed data were grouped based on the nurses’ responses on each technology for each of the five questions. For example, the first question was, “what are the benefits or disadvantages you are experiencing for using smart Beds, dome light indicators, and smart hand hygiene support system”. Their response, on smart beds for example, “it used to be falls every day but now we don’t see many falls in the unit” was grouped under the response for the first questions which is a relative advantage.

## Results

### Patient falls

#### Descriptive analysis

From January 2012 to October 2016, 371 inpatient falls were reported in the MHIU; these were classified into 11 different types: bed/crib, chair/wheel, fainted, lost balance, lowered to the floor during transfer, slipped, toilet/commode, transfer, tripped, tub/shower, and other. A detailed descriptive analysis regarding these types is provided in Table 2. Most of the patients who fell were male (60%) with a mean age of 77.05 years. Of the 68 falls caused by beds (18.3% of the total), 44 occurred during the pre-intervention period (mean rate of 1.27 per quarter) and 24 occurred during the post-intervention period (mean rate of 0.91 per quarter). A statistical analysis using t-test comparing the pre- and post-intervention periods demonstrated no statistically significant change in average falls/quarter rate and average bed falls/quarte rate—however the rate of bed falls declined from 21 to 15% of the total falls following the intervention.

**Table 2:** Descriptive analysis of patient falls

		All	Pre-intervention	Post-intervention	*P*-value
Average falls/quarter rate (t-test)			6.01	5.80	*P* = 0.9
Average bed falls/ quarter rate (t-test)		1.1	1.27	0.91	*P* = 0.26
Average Age		77.05	77.4	76.7	
Gender (male)		222 (60%)	132 (63%)	90 (67%)	
Types of falls	Bed/crib	68	44	24	
	Chair/wheel	45	17	28	
	Fainted	4	2	2	
	Lost balance	121	65	56	
	Lowered to floor during transfer	7	6	1	
	Other	35	29	6	
	Slipped	60	26	34	
	Toilet/commode	18	13	5	
	Transfer/transport	6	3	3	
	Tripped	6	4	2	
	Tub/shower	1	1	0	
	Total	371	210	161	

**Fig. 1:**
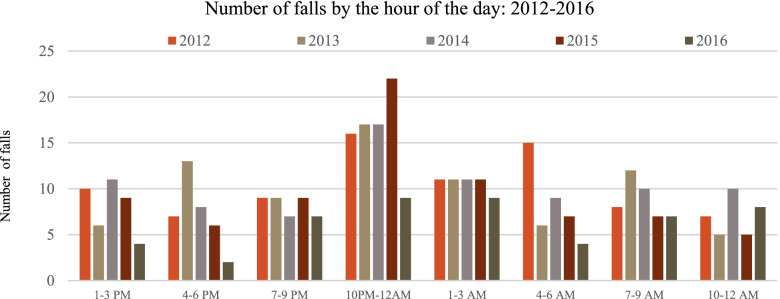
Number of total falls by the time of day

We investigated further to identify the times at which falls are occurring most frequently. Figure 1 illustrates that falls happen during almost every hour of the day; however, 10 p.m. – 12 a.m. featured the highest number of falls.

#### ITS of patient falls

As no statistically significant changes have been found between the pre- and post-intervention periods, we conducted an interrupted times series analysis. Initially, we modeled an ordinary least square regression to detect any change in average patient falls per quarter per 1000 patient days. Though there was a change in trend and slope between the pre- and post-intervention periods, the results were not statistically significant. While checking for serial collinearity with the Durbin-Watson test, we detected serial autocorrelation in the order of lag 1, confirmed by the PACF plot. Therefore, we fit the AR (1) model into our data (Table 3).

**Table 3:** ITS-AR (1) model results for patient bed falls /quarter rate

	Coefficient	p-value	95% confidence interval
Intercept	1.42	0.0005	0.85 to 2.04
Before quarterly variation	− 0.024	0.62	−0.11 to 0.06
Change immediately after intervention	0.5	0.32	−0.41 to 1.42
After intervention	−0.14	0.13	−0.3 to 0.027

**Fig. 2:**
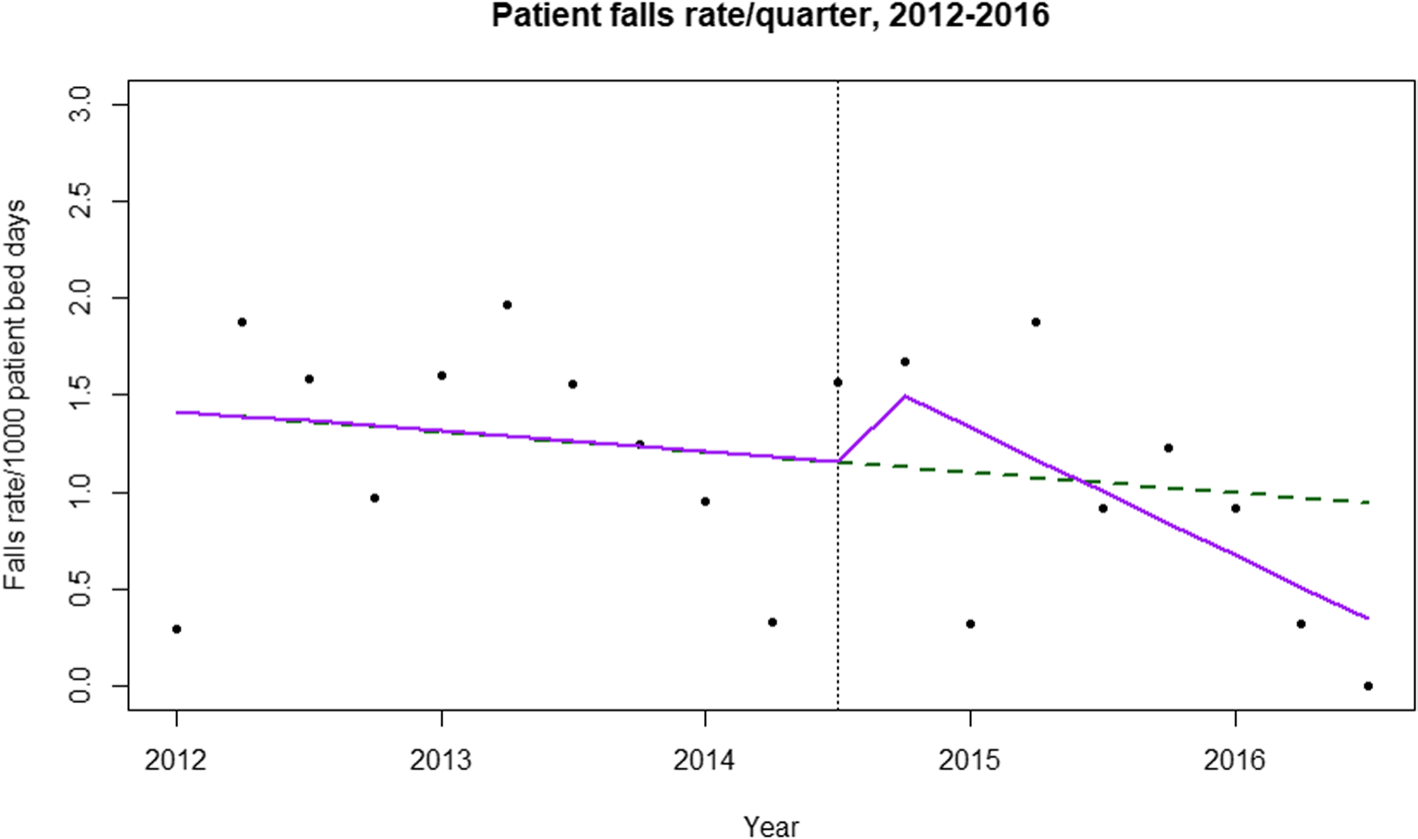
Interrupted time series analysis of patient bed falls. The vertical line separates the pre- and post-intervention periods. The green line estimates the fall rate if there had been no intervention; the purple line shows the true conditions

The results from Table 3, illustrated in Fig. 2, show that (i) during the pre-intervention period, the patient bed fall rate per quarter per 1000 patient days was 1.42; (ii) during the pre-intervention period, the rate was decreasing by an average of 0.024 per quarter; (iii) there was an increase in level (intercept) at the onset of the intervention by 0.5 bed falls per quarter; and (iv) there was an insignificant incremental change in slope (− 0.14 day/month) between the pre- and post-intervention periods.

The dotted line in Fig. 2 estimates the quarterly bed falls rate without the intervention. Though the rate empirically shows a downward trend following the intervention, we could not statistically demonstrate causation due to the limited sample size.

### Hand-hygiene-compliance rate

We examined HH-compliance (handwashing before entering and after exiting a room) rates from September 2014 to October 2016 using only post-intervention data by considering (i) staff type; (ii) room; and (iii) time of day. Our detailed analysis revealed the following:

#### (i) HH-compliance rate by staff type

**Fig. 3:**
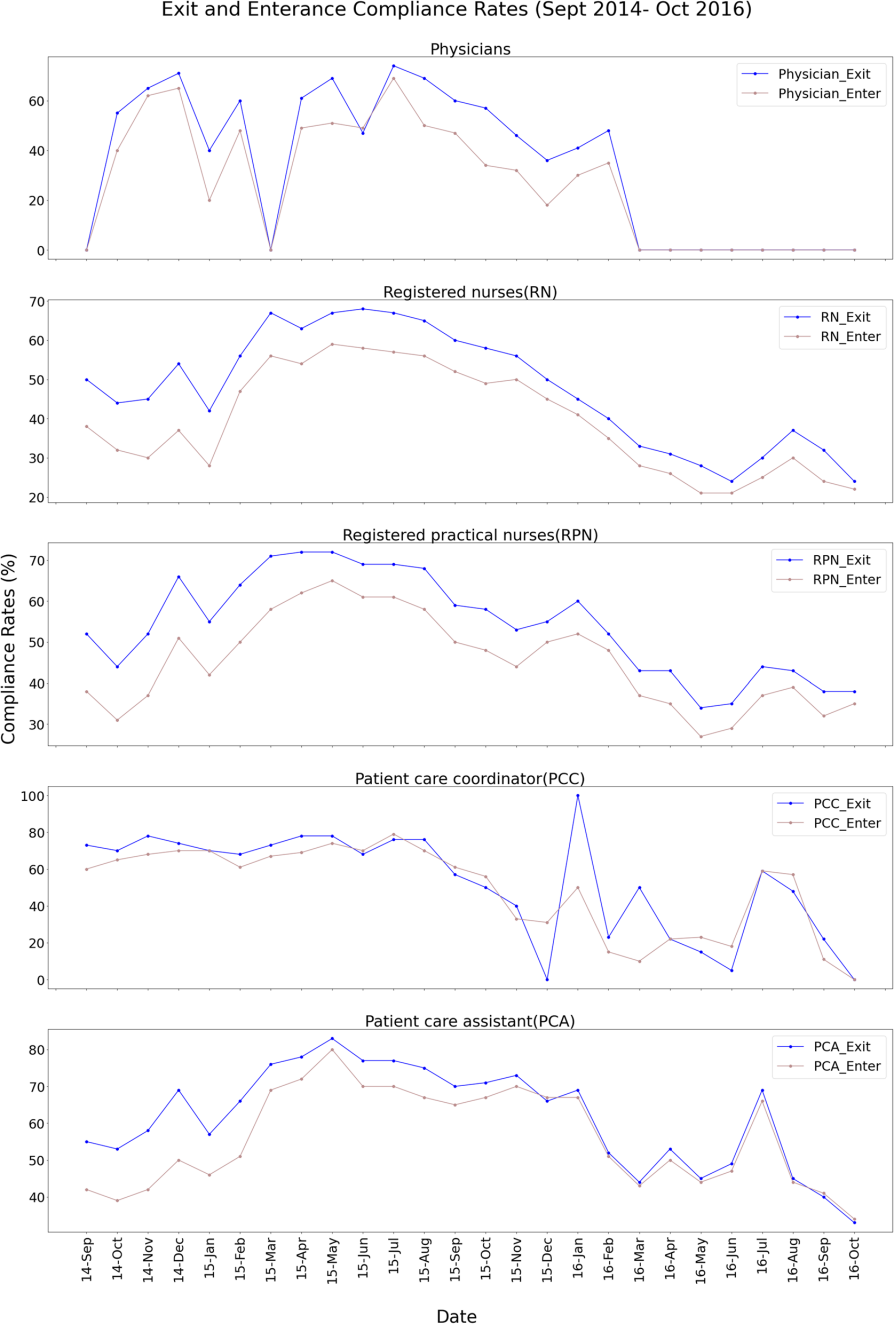
Compliance rate of different staff types

Within the timeframe of our analysis, patient care coordinators (PCCs) had the highest average HH-compliance rate (64%), above physicians (54%), registered nurses (45%), registered practical nurses (48%), and patient care assistants (PCAs) (58%). When we studied the entrance and exit HH-compliance rates separately, we found that—across all staff types—compliance rates were higher during exiting than upon entering. Further analysis revealed that—once more, across all staff types—HH-compliance rates increased from 2014 (creation of the MHIU) to the middle of 2015 before decreasing in 2016, likely due to broken sensors and a lack of awareness among nurses. While nurses initially received daily HH-compliance feedback, that practice was discontinued in mid-2015. Figure 3 shows the HH-compliance rates for all staff types.

#### (ii) HH-compliance rate by room type

**Fig. 4:**
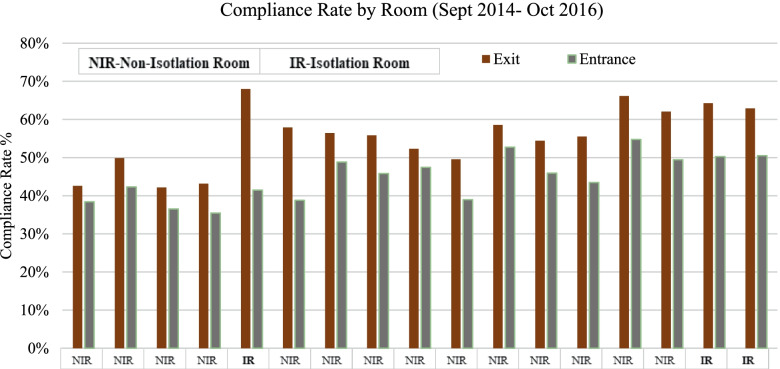
HH-compliance rate by room

Later, we conducted a detailed analysis of the HH-compliance rate for each room in the MHIU. Of the 17 rooms, three were used as isolation rooms (Fig. 4). We found that both entrance and exit HH-compliance rates were higher for isolation rooms than for any other room type over the total study period (2014–2016). Still, further analysis with monthly data confirmed that the exit HH-compliance rate is higher than the entrance HH-compliance rate across all room types; it also confirmed that there was a decrease in HH compliance after 2015.

#### (iii) HH-compliance rate by time of day

**Fig. 5:**
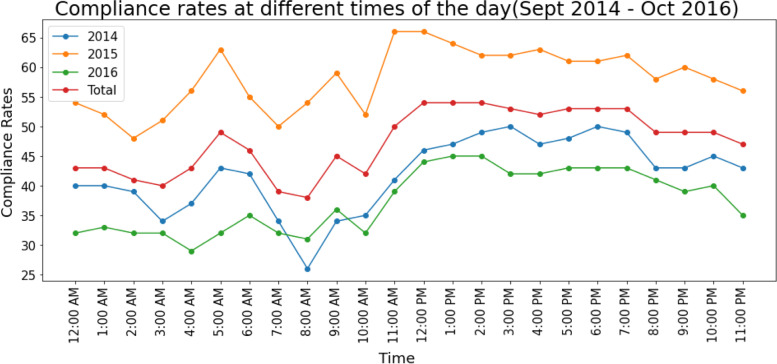
HH-compliance rate by time of day

We analyzed HH-compliance rates at different times of the day to identify the hours during which HH opportunities were most frequently utilized. There were HH spikes at 5 a.m., 9 a.m., and 11 a.m.; these times coincide with unit rounds and other time-based tasks (Fig. 5).

### Staff experience

Based on their responses, the nurses largely agree that smart beds have helped to significantly reduce patient falls and enhance patient care on account of the weighing and quick-inclination features. Nurses also mentioned that the dome light indicators were helpful to achieve fewer falls and locate the proper staff type. They emphasized that, after this intervention, there was a smaller number of falls in the unit. For the HH support system, although most of the nurses were unaware that sensors in the HH dispensers monitored their compliance rates, all of them mentioned that the feedback (which was ultimately discontinued) motivated them to keep their hands clean. Despite all of the advantages, however, nurses also noted the complexities they faced. For example, the beds frequently become unplugged while delivering care; while a light turns on if it’s disconnected, they initially needed training on reconnecting and adjusting all of the bed’s many parameters. Regarding the HH support system, they believe that the timely refilling of the dispensers is important, and that dispenser placement and height are two issues that must be considered.

## Discussion

This paper, to our knowledge, constitutes the first empirical study to assess the impact of IoT interventions on indicators of patient safety, such as patient falls and HH compliance, in a hospital setting. This study examined the effect of IoT intervention (smart beds, HH support systems, RFID badges, dome light indicators, wall-mounted call stations, mobile phones, smart stations) at a hospital unit in Ontario, Canada on patient safety. It highlighted several core messages on IoT implementation in healthcare.

Our study found a reduced number of patient bed falls following the implementation of an advanced IoT-based intervention. This aligns with the literature, which largely asserts that IoT reduces patient falls. Like many other studies, we found that fall incidents were higher among male patients in both the pre- (63%) and post-intervention (67%) periods [63, 64]. We also observed that most of the patients who experienced falls in the MHIU were over 75 years old in both the pre- and post-intervention periods, meaning that the group is relatively vulnerable to injuries; once more, this aligns with the existing literature [65]. Studies have shown that while not all patient falls result in serious injury, the complications associated with falls rise steadily alongside age—they are twice as complicated among patients who are more than 75 years old [65]. Additionally, we found that most of the falls occurred at night (10 p.m. – 12 a.m.); again, this finding was consistent with the findings of most previous studies [66].

While we did not find statistically significant results in our descriptive analysis or ITS study for patient falls, our trend analysis did reveal that the proportion of bed falls has decreased from 21 to 15% following the intervention. Staff interviews also indicated that IoT implementation supported patient safety, improving the quality of care delivery by reducing falls while satisfying the staff. According to the nurses in the unit, alerts from smart beds and dome light indicators significantly contributed to the decline in the number of falls during the post-intervention period. Additionally, the nurses indicated that bed falls notably decreased following the intervention. Of course, as the results are not statistically significant, we cannot conclude that the IoT intervention is the only reason for this change. However, as most of the falls occur at night among older male patients in this unit, the smart bed’s alert system may have had a great impact on this improvement, as suggested by the literature [34–36, 41].

This study found that the average entrance and exit HH-compliance rates in the MHIU were 43 and 54%, respectively, throughout the study period. Interestingly, a similar study found that the HH-compliance rate rose from 67 to 70% following the intervention [67]. Our study shows a range in HH-compliance rates from 45 to 68% across the various staff types (PCC, PCA, physicians, registered nurses, and registered practical nurses). A similar range—26 to 64%—was found in an existing study [68]. Further analysis of staff type revealed that PCCs had the highest HH-compliance rate. We also found that the exit HH-compliance rate was uniformly and consistently higher (54%) than the entrance HH-compliance rate (43%); this finding aligns with previous studies [69]. One potential reason for such a behavior is that the staff may have entered another room immediately exiting another room—meaning they had just cleaned their hands.

Another finding related to hand hygiene was that isolation rooms had the highest HH-compliance rates across all staff types, which—once again—aligns with the existing literature. The average entrance and exit HH-compliance rates for the three isolation rooms in the MHIU over the study period were 47 and 65%, respectively; the same rates for the 14 non-isolation rooms were 44 and 53%. A similar trend was detected in previous studies, where researchers showed that HH activity was 49% more likely in isolation rooms [70]. This makes sense, as the purpose of isolation rooms is to prevent the transmission of microorganisms to the staff or other patients. Staff members follow a specific protocol while delivering care to isolated patients. As already noted in the literature, this extra protection likely contributed to the relatively high HH-compliance rate [70].

Another major takeaway from this study is that proper knowledge of all intervention components among the staff is essential for a successful intervention; this knowledge can be obtained through small training sessions and support during the implementation process. This assessment has also been made in other studies [71, 72]. This study showed that the considered technologies need to be actively maintained and adjusted to strengthen the intervention’s impact. For example, we observed the exit HH-compliance rate at 52% in 2014, then 65% in 2015 but 44% in 2016. Similarly, the entrance HH-compliance rate went from 35 to 56% and 38% in 2014, 2015, and 2016, respectively. Both HH-compliance rates declined starting in mid-2015, partially due to broken sensors and the end of daily feedback. Both of these explanations behind declining HH-compliance rates align with the literature; one study analyzed 1120 survey responses and found broken sensors to be a core issue hampering HH compliance [73]. Using feedback to improve HH compliance has been reported numerous times. Researchers note that individual feedback significantly improves HH-compliance rates; one study found that compliance among nurses increased from 43 to 55% following the provision of individual feedback [74].

Our analysis of the smart beds revealed that a frequent call type that was unrelated to patients was the “bed-disconnected” call. This call alerts caretakers when a bed is unplugged and is very quiet. Therefore, it often goes unnoticed, potentially contributing to patient fall rates.

## Limitations

Our study has some important limitations to note. Regarding the clinical endpoints, such as patient falls, our sample size was quite small due to a lack of data availability. Though studies involving ITS have no minimum data-point requirements, the power of the analysis is increased if the data points are equally distributed across the pre- and post-intervention periods—this was not possible in our case [61]. Our small number of data points skewed toward the post-intervention period may have contributed to the lack of statistical significance. While we observed a declining fall rate and heard comments to that effect from the nurses, we cannot conclude that this stems entirely from the intervention, as we did not consider any other internal (e.g., demographics, clinical information) or external (e.g., time, weather) factors. However, it is worth noting that the reporting of insignificant results is not uncommon in the literature [75]. Our participant number for the interview was small as well. Considering that this intervention was applied only to one unit, it was not possible to recruit a higher number of staff.

Additionally, we could not compare the post-intervention HH-compliance rates with those of the pre-intervention period, as historical HH-compliance rates were manually audited periodically and were inordinately high. Therefore, in line with the literature, we suspect a relationship between manual periodic audits and high compliance rates [76].

We have reported compliance rates across different times of day, staff types, and rooms; however, we did not measure whether this HH support system had any impact on the unit’s infection rates due to a lack of data.

## Conclusion

We conducted a thorough analysis of the impact of the IoT-based interventions on patient safety and found a positive impact on various aspects of patient safety. Though our study could not find any statistically significant changes in the mean patient fall rates, however qualitative interviews with nurses stated reduced patient falls and improvements in direct care time. This study also studied the HH compliance rates, where an increase in the first year was reported, followed by a decrease in the second year. While this study details promising benefits of IoT in patient safety, further analysis that includes recent data on patient falls, HH compliance, and infection rates would provide further findings.

## Data Availability

The authors underwent a vigorous privacy approval from different departments of the hospital to obtain the data. Therefore, it is not publicly available. However, on any reasonable request, the aggregate datasets that have been used during this study can be provided from the corresponding author with approval from the hospital.
